# Evaluating community health worker education policy through a National Certificate (Vocational) Primary Health qualification lens

**DOI:** 10.4102/phcfm.v12i1.2104

**Published:** 2020-02-06

**Authors:** Michelle N.S. Janse van Rensburg, Tessa S. Marcus

**Affiliations:** 1 Department of Family Medicine, School of Medicine, Faculty of Health Sciences, University of Pretoria, City of Tshwane, South Africa

**Keywords:** NC(V) Primary Health, Community Health Worker, PHC Re-Engineering, Education and Training, WHO Guideline, Ward-Based Outreach Teams

## Abstract

**Background:**

In 2018, the South African National Department of Health (NDoH) published a 5-year policy framework and strategy for Ward-Based Primary Healthcare Outreach teams to improve team management and leadership and support service delivery. In the same year, the World Health Organization (WHO) published guidelines on health policy and system support to optimise Community Health Worker (CHW) programmes.

**Aim:**

This article aims to assess the National Certificate (Vocational), or NC(V), Primary Health qualification in terms of the education and training guidelines and recommendations of the 2018 NDoH and WHO policy documents.

**Setting:**

The qualification was initiated in 2013 at 12 Technical and Vocational Education and Training (TVET) colleges across South Africa. The evaluation covered the period 2013–2017.

**Methods:**

Pragmatic qualitative enquiry was used to examine the context, design, implementation and outcomes of the qualification. Data collection involved document reviews, key informant in-depth interviews and focused group discussions, and individual reflections with respondents from one part-time and two full-time offerings at two colleges. Analyses of emergent themes were interpreted using appropriate models and theoretical frameworks.

**Results:**

The Department of Higher Education and Training (DHET) created and implemented a standardised, curriculated national programme for CHW education that structured theoretical and practical learning over time to ensure assimilation of content and its application in practice.

**Conclusion:**

NC(V) Primary Health, as a single, national, quality-assured qualification for CHWs, meets WHO 2018 guidelines and recommendations, NDoH training needs and CHWs learning expectations, especially when offered part-time. Despite the termination of the programme, it remains a relevant option for CHWs in South Africa and elsewhere.

## Introduction

South Africa’s transition to a constitutional democracy over the past 25 years has witnessed considerable progress in the reversal of apartheid discriminatory practices.^1^ Even so, the health and well-being of the majority of South Africans remain burdened by persistent social disparities, infectious and non-communicable diseases, the scourge of violence and injury, and inadequate human resources to provide health and care to a growing population.^1^

There is global and national consensus that these challenges can only be adequately addressed through universal healthcare coverage and prioritisation of the social determinants of health.^1^ In South Africa, this is evident in the 2010 Primary Health Care Reengineering (rPHC)^2,3,4^ policy, intended to strengthen health promotion, disease prevention and early disease detection^2^ through the introduction of district specialist teams, school health teams^5^ and Ward-Based Outreach Teams (WBOTs)^3,4^ – subsequently termed Ward-Based Primary Health Care Outreach Teams (WBPHCOTs).^6^ Also, the National Development Plan (NDP) 2030 Vision, published in 2012, envisages that population health and well-being will be achieved through a strengthened health system^7^ where ‘each household must have access to a well-trained community health worker’ (Goal 7).^8^

Implementation of rPHC began in 2011 and WBOTs were initiated in nine National Health Insurance (NHI) pilot sites in seven provinces in 2012.^3^ Approaches to WBOTs have been varied during the first 7 years of implementation.^5^ Each province interpreted the initiative differently, with Gauteng, for example, explicitly committing to Community-Oriented Primary Care (COPC).^9,10^ The provinces faced common challenges, such as poor preparatory planning, including a failure to include WBOTs in annual financial plans^3^; a lack of clarity about the place of Community Health Workers (CHWs) and community-based services in the healthcare system, and a lack of clarity regarding CHW roles and scope^2,11^; difficulties in redefining relationships between NGOs and PHC facilities^9,11^; poor communication about WBOTs within PHC services and between PHC services and communities^5,9^; little or no integration with other streams of rPHC^8^, insufficient political support^9^; problems with supervision and management of CHWs^3,5,11^; inadequate training of CHWs^5,11^; under-provision of equipment and supplies^3,11^; poorly constituted teams^3^; inappropriate payment systems and inadequate levels of remuneration^9^; and a high turnover of CHWs.^11^

In an attempt to address these challenges, in April 2018, the South African National Department of Health (NDoH) published a 5-year policy framework and strategy for the renamed WBPHCOT programme.^6^ It aims to improve the health and well-being of individuals, households and communities through a strategy of community participation, empowerment, intersectoral collaboration and context-specific implementation.^6^ It set out four goals – to improve working conditions (Goal 1); to improve human resource recruitment, selection, placement, development and management (Goal 2); to standardise the scope of work and programmatic application across all nine provinces (Goal 3); and to improve and maintain a monitoring and evaluation system (Goal 4). It also stipulates particular requirements, explicitly mentioning the need for uniform terms and conditions of employment, including that CHWs have Grade 12; the use of specially trained enrolled nurses as outreach team leaders (OTLs); and the need for team performance flexibility with respect to the number of households covered in order to take account of locality variations in travel time and distance. Notably, however, other than being implicit in human resource development (Goal 2), the document has no specific training goals, and references to training are limited to acknowledging the need for training, the assignment of training as a provincial and district responsibility, and the requirement that OTLs should be provided with unspecified ‘special training’.

In October 2018, shortly after the South African policy framework and strategy release, the World Health Organization (WHO) published its guidelines on health policy and system support to optimise CHW programmes.^12^ Drawing from global evidence about the role, potential and challenges of getting CHWs to help respond to pertinent healthcare challenges, the WHO document identifies three overarching issues: (1) CHW selection, training and certification, (2) CHW management and supervision and (3) CHW service integration into health systems and community support.^13^

While the contribution of CHWs to health outcomes depends on many factors, the quality of the services they provide and their potential impact are directly related to their education and training. In South Africa, there is considerable variation in the range and depth of CHW knowledge, skills and competencies.^14^ Previous short courses and certificate programmes were often driven by vertical programmes, tangentially linked to the complexity of healthcare services in the community and were poorly articulated to employment and further education and training opportunities.

Paradoxically, the omission of education and training from the 2018 NDoH policy and strategy framework document is contrary to its clear recognition in 2010 that led to the development and implementation of the National Certificate (Vocational), or NC(V), Primary Health qualification. Positioned within the national vocational certificate training structure designed to provide post-school education in various vocational areas, it was the first ever full-time, 3-year certified qualification at National Qualification Framework (NQF) levels 2, 3, and 4 for CHWs.

In 2011, at the request of the NDoH, the National Department of Higher Education and Training (DHET, appointed curriculum writers and principles were agreed upon regarding the programme name, topics, subject outcomes, learning outcomes and assessments standards per subject and per level (DHET Memo 22, March 2011).

The NC(V) Primary Health programme comprised seven subjects. Four were vocation specific, namely, the South African Health Care System, Public Health, Human Body and Mind, and COPC. Three subjects – First Additional Language, Mathematics or Mathematical Literacy, and Life Orientation – were compulsory across all vocational qualifications. The first NC(V) Primary Health cohort of 1200 full-time students were enrolled in 2013 at 12 Technical and Vocational Education and Training (TVET) colleges on 14 campuses across the country. The qualification was formally terminated in 2016 for reasons unrelated to student performance, the levels of learning and the potential value of graduates to rPHC.

This article provides an assessment of the NC(V) Primary Health qualification in terms of the education and training policy guidelines and recommendations set out in the respective NDoH^6^ and WHO^12^ 2018 documents.

## Research methods and design

The original research aimed to contribute to the discourse around CHW training and education by evaluating the NC(V) Primary Health qualification in the context of health system reform.^15^ Using Stufflebeam’s^16^ context, input, process, product (CIPP) model to structure a qualitative pragmatic enquiry, the study examined the context of health system reform in South Africa and the ‘fit’ of the NC(V) Primary Health programme, how the qualification was put together and implemented, as well as the outcomes.

Beginning 2013, NC(V) Primary Health was offered at 12 TVET colleges across the country. Students were expected to enrol full-time and to cover the standard curriculum over 3 years. In 2014, a part-time offering was piloted in one sub-district of Tshwane. Designed to support the vocational education of employed CHWs working for the City of Tshwane (CoT), candidates were expected to progress through the curriculum over 4 years. Enrolled at an accredited TVET college and taught by lecturers from the Department of Family Medicine (UP), the two cohorts of CHW students active in the study period (see Table 1) worked and studied in Mamelodi.

**TABLE 1:** Student numbers for the first and second cohorts of the part-time offering.

	Cohort 1		Cohort 2	
	Beginning End		Beginning End	
2014	35	18	-	-
2015	18	17	-	-
2016	16	16	29	20
2017	16	16	20	16
2018	6	4	14	14
2019	4	-	14	-

*Source*: Compiled by the authors from Department of Family Medicine records.

All TVET colleges in South Africa that presented NC(V) Primary Health were approached to participate in the study. Only two agreed on condition that interviews should be confined to college management and lecturers. Their decision to prohibit the participation of full-time students arose from ongoing uncertainty about the future of the programme and student disquiet about the lack of articulation to employment in healthcare.

Primary data were collected from a total of 65 respondents. Thirteen key informant interviews were conducted with relevant role players from the health and education sectors, including curriculum experts, a consultant to the NDoH, a WBOT team leader, academics involved in frontline and mid-level worker education and college management from both TVETs. Two focus group discussions were conducted with full-time lecturers at each of the respective TVET colleges and six focus group discussions were held with part-time CHW students. Written reflections were obtained from full-time students (*n* = 9) at one TVET college and from the part-time offering lecturers (*n* = 5). Additional primary data included student assessments, minutes of meetings and field notes. Secondary data were derived from policies, reports, subject guides, textbooks and manuals.

Interviews and focus groups were conducted in English, audio-recorded and transcribed. Both inductive^17^ and deductive^18^ analyses were conducted to discover emergent themes and to interpret the data by applying appropriate models and theoretical frameworks to transcriptions and document reviews. Creswell and Poth’s data analysis spiral^19^ was followed, which is used to identify patterns and relationships^20^ by reading, memoing and reflecting on the data to develop emergent ideas, classify them into themes, develop and assess interpretations, and review accounts of the findings to ensure representation, accuracy and authenticity.^19^ The data were positioned in terms of the CIPP model’s context (the history and background of the programme, the defined goals, as well as needs, problems, assets and opportunities), inputs (financial, material, time, physical and human resources needed for the programme to work effectively and achieve its goals), process (the implementation of the programme) and product (the intended, unintended, short-term and long-term outcomes of the programme and its potential usefulness or benefit to individuals and society).^16,21^

Trustworthiness was ensured in several ways. There had been engagement with the qualification from inception throughout implementation, through content and assessment development, as well as national TVET lecturer training for each of the respective levels. Peer debriefing with lecturers assisted researcher interpretation and understanding. Data triangulation was used to support reliability, given the multiplicity of data sources. Thick descriptions were used to support transferability within the domain of CHW education. The research followed a logical and well-documented process, including reflexive note taking to enhance dependability. Lastly, the researcher engaged with national and international leaders in health science education, primary healthcare and frontline healthcare worker training to ensure the confirmability of ideas and arguments.^22^

Informed consent was obtained from each participant along with written permission to record focus groups and key informant interviews. While anonymity cannot be guaranteed during focus groups, confidentiality was adhered to as no names of individuals were recorded in the collection of data or made known in the reporting of information.

### Ethical considerations

Data analysis was conducted during and after collection. Ethical approval for the research that informs this study was obtained from the University of Pretoria’s Faculty of Health Sciences’ Ethics Committee (protocol number: 46/2015).

## Results

The information obtained from the CIPP evaluation of the NC(V) Primary Health qualification are juxtaposed against the education and training goals and objectives of the NDoH policy framework and strategy, and matched and aligned with the WHO recommendations pertaining to the selection, training and certification of CHWs (see Table 2).

**TABLE 2:** Comparison of the World Health Organization policy recommendations and the National Department of Health Policy Framework and strategy for Ward-Based Primary Health Care Outreach Teams.

WHO recommendations		Policy framework and strategy for WBPHCOTs 2018/19-2023/24
Selecting, training and certifying CHWs†		Goal 2: Improve human resource recruitment, selection, placement, development and management
		Goal 3: Standardise the WBPHCOT scope of work and ensure standardised application
1. Selection	Specify minimum educational levels. Require community membership and acceptance. Consider personal capacities and skills. Apply appropriate gender equity to context.	Objective 7: Ensure appropriate implementation and management of recruitment, selection … processes for all members of WBPHCOTs.
2. Pre-service training duration	Base on CHW roles and responsibilities. Consider pre-existing knowledge. Factor in institutional and operational requirements.	Objective 7: Ensure appropriate implementation and management of recruitment, selection, appointment, placement, remuneration, skills development, dispute resolution and occupational health and safety processes for all members of WBPHCOTs. Objective 10: Confirm training content and method for ensuring the WBPHCOTs are capacitated to provide required services.
3. Curriculum to develop competencies	Train on expected preventive, promotive, diagnostic, treatment and care services. Emphasise role and link with health system. Include cross-cutting and interpersonal skills.	Objective 2: Standardise roles and responsibilities of … CHWs in the provision of community-level services. Objective 7: Ensure appropriate implementation and management of recruitment, selection, appointment, placement, remuneration, skills development, dispute resolution and occupational health and safety processes for all members of WBPHCOTs. Objective 10: Confirm training content and method for ensuring that WBPHCOTs are capacitated to provide required services.
4. Training modalities	Balance theory and practic. Use face-to-face and e-learning. Conduct training in or near the community.	Objective 10: Confirm training content and method for ensuring that WBPHCOTs are capacitated to provide required services.
5. Offer competency-based formal certification upon successful completion of training	-	Objective 9: Ensure standardised implementation of the approved scope of work. Objective 10: Confirm training content and method for ensuring that WBPHCOTs are capacitated to provide required services.
**Managing and supervising CHWs**		**Goal 1: Improve working conditions of WBPHCOTsGoal 2: Improve human resource recruitment, selection, placement, development and management**
6. Supportive supervision	Establish appropriate supervisor-CHW ratios. Train and resource supervisors to provide meaningful, regular performance evaluation and feedback. Use supervision tools, data and feedback to improve quality. Use supervision tools, data and feedback to improve quality.	Objective 1: Standardise the WBPHCOTs’ management structures at provincial and district levels. Objective 2: Standardise roles and responsibilities of … clinic manager, … CHW team leader … in the provision of community-level services. Objective 7: Ensure appropriate implementation and management of recruitment, selection, appointment, placement, remuneration, skills development, dispute resolution and occupational health and safety processes for all members of WBPHCOTs. Objective 8: Ensure adequate supervision and support for CHWs as well as for WBPHCOT leaders.
7. Remuneration	Include resources for incentives in health system resource planning. Provide a financial package commensurate with the job demands, complexity, number of hours, training and roles that CHWs undertake.	Objective 3: Complete the CHW investment case to obtain the required budget over the Medium Term Expenditure Framework (MTEF) period for a well-resourced and well-functioning institutionalised CHW programme. Objective 7: Ensure appropriate implementation and management of recruitment, selection, appointment, placement, remuneration, skills development, dispute resolution and occupational health and safety processes for all members of WBPHCOTs.
8. Contracting agreements	For paid CHWs, establish agreements specifying roles, responsibilities, working conditions, remuneration and workers’ rights.	Objective 4: Complete and maintain the national CHW information database and use the information to confirm existing CHWs in teams required to serve specific communities. Objective 7: Ensure appropriate implementation and management of recruitment, selection, appointment, placement, remuneration, skills development, dispute resolution and occupational health and safety processes for all members of WBPHCOTs.
9. Career ladder	Create pathways to other health qualifications or CHW role progression. Retain and motivate CHWs by linking performance with opportunities. Address regulatory and legal barriers.	-
**Integrating into health systems and gaining community support**		**Goal 2: Improve human resource recruitment, selection, placement, development and management** **Goal 3: Standardise the WBPHCOT scope of work and ensure standardised application**
		**Goal 4: Improve and maintain the monitoring and evaluation system for the WBPHCOT programme**
10. Target population size	Consider population size, epidemiology and geographical and access barriers. Anticipate expected CHW workloads, including nature and time requirements of the services provided.	Objective 5: Define an adequate ratio of WBPHCOTs to population and households allowing for differential geographic distribution, and considering problems with access in rural areas. Objective 6: Ensure that WBPHCOTs are fully staffed and equitably distributed throughout South Africa.
11. Collection and use of data	Enable CHWs to collect, collate and use health data on routine activities. Train CHWs and provide performance feedback based on data. Minimise reporting burden, harmonise requirements and ensure data confidentiality and security.	Objective 12: Review and standardise current indicators and data collection tools across all provinces. Objective 13: Establish the required structures at national, provincial, district and PHC facility levels for data collection and reporting. Objective 14: Ensure submission of monthly activity data from PHC facilities to the District Health Information System (DHIS), quarterly progress reports, as well as a 5 yearly outcome and impact reports from NDoH and provinces.
12. Types of CHWs	Adopt service delivery models comprising CHWs with general tasks as part of integrated primary health care teams. CHWs with more selective tasks to play a complementary role based on population health needs, cultural context and workforce configuration.	Objective 5: Define an adequate ratio of WBPHCOTs to population and households allowing for differential geographic distribution, and considering problems with access in rural areas. Objective 6: Ensure that WBPHCOTs are fully staffed and equitably distributed throughout South Africa. Objective 9: Ensure standardised implementation of the approved scope of work.
13. Community engagement	Involve communities in selecting CHWs and promoting programme use. Engage relevant community representatives in planning, priority setting, monitoring, evaluation and problem-solving.	-
14. Mobilisation of community resources	CHWs to identify community needs and develop required responses. CHWs to engage and mobilise local resources. CHWs to support community participation and links to health system.	Objective 11: Ensure, as part of the Ideal Clinic programme, that WBPHCOTs have adequate physical space in clinics to prepare for their day in the field and to meet their data recording and reporting responsibilities.
15. Supply chain	Ensure CHWs have adequate and quality-assured commodities and consumables through the overall health supply chain. Develop health system staff capacities to manage the supply chain, including reporting, supervision, team management and mHealth.	-

Source: Compiled by authors by comparing the WHO guideline for CHW programmes^13^ and the NDoH WBPHCOT policy and strategy framework.^6^

WBPHCOT, Ward-based Primary Health Care Outreach Teams; CHW, community health worker; NDoH, National Department of Health; PHC, primary health care.

† , This article focuses on the first five recommendations, which pertain to selecting, training and certifying CHWs.

### Selection

In terms of selecting CHWs, the WHO recommendations state that CHW programmes should specify minimum educational levels, require community membership and acceptance, consider personal capacities and skills, and apply appropriate gender equity to context.^13^ The NDoH policy emphasises the importance of improving human resource recruitment, selection, placement and development (Goal 2). It states that the ‘minimum requirements for CHWs should be matriculation (Grade 12), subject to training programmes’ and that recognition of prior learning principles should ‘be applied to CHWs who are already in the system and who have undergone relevant training’, where possible.^6^ In addition, it states that appropriate implementation should be ensured (Objective 7) and stipulates the implementation responsibilities of the different levels of the health system. Provinces are made responsible for ensuring that training ‘for new and existing members of WBPHCOTs are [*sic*] in place’ and districts are required ‘to develop, implement and maintain a capacity building system for all CHW team members within a multidisciplinary team context’.^6^

The NC(V) Primary Health qualification is designed as a parallel vocational pathway for schoolgoers. It does not require Grade 12 and there are certified exit possibilities at each level. Learners who have completed Grade 9 schooling can enrol in the programme. In terms of the National Qualifications Framework (NQF), NC(V) level 2 is equivalent to Grade 10, level 3 is equivalent to Grade 11 and level 4 is equivalent to the National Senior Certificate (Grade 12).^23^ The evaluation found that several full-time students and all the part-time NC(V) CHW students had Grade 12, with one having a post-school diploma. Although seemingly paradoxical, students with Grade 12 saw the NC(V) as an opportunity for a second chance and accepted repeating levels of learning, albeit with some reluctance. Mostly they said they did this because it provided them with an opportunity to improve their English and Mathematics, and it was ‘better’ than Grade 12, especially in terms of the depth of the vocational subjects (In-depth interview, December 2016).

Regarding the value of prior learning, part-time students especially brought a great deal of knowledge into the learning environment as they were practicing CHWs and many had a history of working in communities through non-governmental organisations (Expert interview, 09 September 2016).

With respect to community membership, NC(V) Primary Health students in both the full-time and part-time offerings, for the most part, were drawn from and lived in communities in the vicinity of the colleges or campuses where the programmes were offered. They, however, were not selected by local community structures as is the practice in other settings in Africa and elsewhere. Rather, students applied volitionally to study through a learning system that is open to everyone. In so doing they had to demonstrate an interest in the course and write a numeracy and literacy entrance examination. Full-time students were also expected to contribute nominally towards the costs of their studies or secure funding from the National Student Financial Aid Scheme (NSFAS) where this was possible. Part-time students were exempt from paying fees as it was a pilot project involving CHWs earning below the minimum wage. The NC(V) Primary Health programme as a whole was substantially subsidised financially by the Health and Welfare Sector Education and Training Authority (HWSETA).

Regarding gender equity, annually about 90% of NC(V) Primary Health students were females.^24^ Although the predominance of females among the students reflects the gendered nature of the health and care workforces in South Africa and elsewhere,^25^ men do work in primary health care and are enrolled in health care training. The training and work raise men’s understanding of their own bodies and health, and it gives them purpose (Focus Group, 19 January 2017). Also having male CHWs as part of the WBOT makes contact with hard-to-reach men and boys at risk easier.

### Pre-service training

The NC(V) Primary Health programme development was guided by the rPHC understanding that ‘interim on-the-job orientation, training and skills development will be offered to increase the capacity of community-based health workers to fulfil the responsibilities of CHWs’^26^, but that the NC(V) Primary Health initiative would also offer CHWs an opportunity to acquire a qualification that fitted in the NQF and was quality assured. The curriculum was based on the rPHC scope and roles of CHWs outlined in the 2011 provincial WBOT implementation guidelines. These were to:

- promote health and prevent illness
- conduct structured household assessment to identify their health needs
- provide psychosocial support to community members
- to conduct community assessments and mobilise around community needs
- to identify and manage minor health problems
- support the continuum of care through service coordination with other relevant  service providers
- support screening and health promotion programmes in schools and Early Childhood Development (ECD) centres.^26^

While the WHO guidelines state that pre-service training should be based on CHW roles and responsibilities, and the 2018 NDoH policy expands somewhat on the 2011 scope, the latter provided no guidance on content and method.^6^

In terms of duration, the NDoH interim Phase 1 pre-service training was implemented over 10 days of continuous contact, while the NC(V) Primary Health programme extended over 3 years, full-time, in accordance with existent DHET stipulations. Recognition that full-time, college-based learning with little workplace exposure was not appropriate to learners’ and service providers’ needs, the DHET piloted a part-time NC(V) Primary Health offering with the Gert Sibande TVET college and the Department of Family Medicine at UP and the CoT. As the course was offered over 4 years, CHWs were able to continue to work in CoT WBOTs and study for the qualification. Many of the CHW students enrolled part-time persisted to level 4 (see Table 1), even though it took them longer to qualify. For them, the length of time to qualification was significantly less important than healthcare system failure to recognise their qualifications in terms of value to the service and career progression.

The NC(V) Primary Health is one of 17 DHET NC(V) programmes. Curricula for all are underpinned by the NQF, the objectives of which are to create an integrated national framework for learning achievements; facilitate access to and progression within education, training and career paths; enhance quality; redress unfair discrimination and past imbalances; accelerate employment opportunities and contribute to the holistic development of students.^27^

All programmes are guided by structured, clearly set out guidelines and templates for course implementation and assessment that derive from national policy on the conduct, administration and management of NC(V) assessments (DHET, Internal Continuous Assessment [ICASS] guidelines for NC(V) qualifications, 2015–2018). These are provided to TVET college course administrators and lecturers to ensure uniformity of planning, presentation, assessment, record-keeping and reporting. Tests, examinations and practical assessments for vocational subjects are quality assured through moderation, monitoring and verification. Student performance in all programmes is also assessed through a national, standardised end-of-year external examination for all subjects, as well as through Integrative Summative Assessment Tasks (ISATs) for vocational subjects. Annually, the quality and standard of all NC(V) assessments and their compliance to policies and guidelines are evaluated by Umalusi and the DHET.^28^ These processes and practices ensure the credibility of NC(V) qualifications.

Practical assessments are central to the vocational nature of the NC(V) qualification as they require integration of theory and practice. The Practical Assessment Tasks (PATs) from the ICASS, along with the ISATs, require students to demonstrate that they can apply theoretical knowledge to practical scenarios that simulate real-life situations. These assessments can thus measure a graduate’s readiness for employment.

### Curriculum to develop competencies

The NC(V) Primary Health curriculum was constructed to align competencies with NQF levels. These guide competence and achievement across qualifications to ensure that learning is consistently and appropriately situated and nationally and internationally comparable.^29,30^ Thus, with respect to problem solving, for instance, at level 2 a learner is required to demonstrate an ability to use his or her own knowledge to select and apply known solutions to well-defined routine problems. At level 3, a learner should be able to use his or her own knowledge to select appropriate procedures to solve problems within given parameters. At level 4, a learner should show the ability to solve common problems within a familiar context and be able to understand the consequences of the adjustments made.^29^

Part-time CHW-students expressed the value of this alignment in various ways (see Table 3). They said that they feel well-equipped to do their work and are better equipped to provide education in households than their colleagues who had not done NC(V) Primary Health. They mentioned that their knowledge, skills and competencies have grown their confidence, and they feel they have a qualification that they and others regard as legitimate and worthy. In addition, they said they feel that people’s confidence in the services they provide has increased. These perceptions were validated by lecturers and programme managers, who commented positively on students’ understanding of community-oriented primary healthcare, anatomy and physiology, as well as their ability to apply their learning to families, neighbours and other community members. Curriculum alignment with NQF also provided CHW students the opportunity to study further and articulate to higher education options.

**TABLE 3:** Community health worker student experiences of curriculum and competency alignment.

Reference	Issue
**Train on expected preventive, promotive, diagnostic, treatment and care service**	
Focus group, October 2018	‘Community health workers can help bridge the gap on tuberculosis by finding clients not on treatment, promoting treatment adherence support for those on treatment and their families. CHWS can also refer clients presenting with TB symptoms to health facilities for further testing. This training has really helped me.’
Focus group, January 2017	‘People from my place come to talk to me. They ask me for help.’
Focus group, November 2016	‘I learnt more things, like living a healthy lifestyle and how to teach people on how to live a healthy lifestyle. And how to take of care of other people outside there, especially patients…[*sic*]’
Focus group, February 2016	‘As a community health worker, my role is to promote and prevent. Then in COPC I have learnt my role and I have learnt the principles of COPC and I have learnt the different types of families in our communities and I have learnt to work with people.’
**Cross cutting and interpersonal skills**	
Focus group, January 2017	‘…When you go there, out there, they ask you a question and then you don’t know it, sometimes you come here and you go to the ICT; it helps us to research sometimes. So, it makes it easier for us because we go there and then we research. So, if it wasn’t for ICT, we won’t be able to research something [*sic*]…’
Focus group, July 2016	‘…I can help my team leader to use the laptop because my team leader, she didn’t know how to use laptop. Because of me doing ICT I can able to help her with her laptop and it makes my gadget cell phone to be simpler [*sic*]…’
Focus group, November 2016	‘In terms of my own life…? I think now my future is bright. I can say that … because I have maths, I can apply to do something…’

*Source*: Compiled by authors from focus group transcriptions.

CHW, community health worker; ICT, information communication technology; COPC, community-oriented primary care.

Several students with Grade 12 performed well enough in the NC(V) Primary Health to qualify for NQF level 5 studies in disability, nursing, social work and the clinical associate programme.

The NC(V) Primary Health is the only health offering at TVET level. The programme was purposively developed by the DHET at the request of the NDoH and HWSETA to support rPHC. In the 3 years prior to level 2 implementation in 2013, the DHET appointed curriculum writers, invited expressions of interest from TVET colleges (termed Further Education and Training colleges prior to 2014) and engaged in a detailed preselection process with potential implementing colleges.

The 12 selected colleges were provided with equipment, given a resource list (DHET, Resource list for NC(V) programmes, 2013), given guidance on lecturer recruitment and were assisted to create practical demonstration rooms. In 2012, they were visited by DHET, NDoH and HWSETA representatives to determine institutional readiness to offer the programme at level 2. Beginning in 2013, a bi-annual training programme was run to orient and prepare programme lecturers for each level of the qualification as it came on stream as well as to provide follow-up and assessment support (NC(V) Primary Health Lecturer Training Report, July 2014). The DHET also translated assessment standards into national examinations.

The vocational subjects were identified by an expert panel of healthcare practitioners for this qualification. These were translated into curricula, with explicit subject and learning outcomes for each level of learning (DHET, NC(V) subject and assessment guidelines for vocational subjects, 2013–2015) and relevant materials^31,32,33,34,35,36,37,38,39,40,41,42^ to support the training.

The NC(V) qualification requires all students to take language (mostly English), life orientation and mathematics or mathematical literacy. The language programme is intended to help students develop or improve their ability to speak and write in English, the lingua franca of official communication. Life orientation has a strong Information Technology (IT) component that equips students to navigate the Internet, as well as to use MS Word, Excel and Power Point for work and study. In it, students are also taught planning, communication, problem-solving and interpersonal skills – all of which are essential for effective healthcare delivery.

With respect to mathematical literacy or mathematics, several TVET colleges offering NC(V) Primary Health worked on an assumption that mathematical literacy sufficed for the level of envisaged work. Others, including in the part-time pilot, offered mathematics, both to support students who aspire for higher education and to use the subject to build CHW reasoning, critical thinking, analytic and interpretation skills (Interview, TVET manager, 07 December 2016).

The extent to which cross-cutting subjects are valued was evidenced by part-time students’ unwillingness to be credited for prior learning, where they had these subjects at Grade 12 level. Amongst other things, they commented that ‘English was very poor the way we were taught in school’, ‘the content is at our level’ and ‘we are gaining more information than when we did matric [grade 12] – it’s more vocationally focused’ (Programme Coordinator Report on NC(V) Primary Health: Part-time Mamelodi-GSC offering, 2015).

### Training modalities

As advocated in the WHO guidelines, training modalities in NC(V) Primary Health sought to balance theory and practice, and face-to-face and e-learning. Training was conducted in or near the community in the part-time offering and, as far as was practically possible, in the full-time offering where the fixed nature of the college infrastructure has to be taken into consideration. Electronic and online educational platforms were used from the onset of the programme to foster collaboration between colleges and lecturers and to support student learning. Part-time students benefitted from contact both in the classroom and in the homes. In the former they were able to clarify ideas, while in the latter they were able to put theory into practice during household visits.

The selection of lecturers from different disciplines was regarded to be an advantage to the programme and to the learners, as it exposed everyone to different methods, approaches and practices (Part-time offering lecturer, written reflection, September 2017).

### Offer competency-based formal certification upon successful completion of training

The NC(V) Primary Health programme by design was intended to create a certified learning pathway from level 2 to level 4. Some 1000 students had graduated at level 4 across the country by 2017 (see Table 4).

**TABLE 4:** NC(V) Primary Health national examination results 2013–2017.

Number and percentage of students who passed										
NC(V)	2013		2014		2015		2016		2017	
			student pass rate		student pass rate		student pass rate		student pass rate	
Level 2	c/a 800 i.	61% ii.	*n* = 693	50.40%	*n* = 830	56.30%	*n* = 994	63,20%	*n* = 279	66.10%
Level 3	n/a	n/a	*n* = 456	63.60%	*n* = 500	60.30%	*n* = 637	68,20%	*n* = 477	77.90%
Level 4	n/a	n/a	n/a	n/a	*n* = 291	56.40%	*n* = 305	56,10%	*n* = 408	61.00%

Source: Compiled by authors from NC(V) records provided by the DHET.

1. It has been difficult to obtain the exact number of candidates who appeared for the 2013 NC(V) Primary Care L2 examination. In NDoH-DHET Human Resource Development Workshop 21 May 2013 meeting notes L2 enrolment was said to be 1200. It is estimated that 75% of students appeared for the examination.

2. DNA Economics (2015) Performance and Expenditure Review: Technical and Vocational Education and Training. National Treasury.

## Discussion

The WHO guidelines are comprehensive in terms of what is required to optimise CHW programmes, while the NDoH policy document of 2018 focuses mostly on the management and leadership of the service delivery aspects of WBOTs. As a consequence, the former is clear and explicit on the selection, training and certification of CHWs, while only some of these elements are explicit in the latter.

In terms of selection, the NDoH policy document stipulates Grade 12 as a minimum educational standard. Following from the WHO recommendation, the standard of entry needs to be carefully considered in the context of the South African education system. The NC(V) Primary Health was created specifically because the schooling system does not adequately prepare many learners for the workplace or further education. This research shows that a vocational training qualification that begins prior to NQF level 4 is an important way of developing the necessary competencies required for CHWs regardless of their level of schooling. It also adds value to prior learning and creates opportunities for further education.^5^

Gender is also an important consideration in selection. The predominance of female CHWs reflects the restricted and gender-biased nature of the South African labour market.^25^ That said, and given the important role of women in the provision of care in South African society, community health work provides an opportunity to be employed and to develop skills through structured learning. There is obviously value to employ male CHWs, as they are possibly more able to meet the healthcare needs of boys and men who are underserved by facility-based services, particularly who are at risk for harmful substance use, HIV, TB and non-communicable diseases.^43^

The WHO recommends pre-service training, that is, education that occurs before being able to do the work and that is sufficient in level, content and duration (not too short and not too long). The NDoH offers 10-day training. This is, by definition, a short course rather than a learning programme. Conceptualised as an interim measure to orientate CHWs and team leaders to WBOTs,^26^ like all short-course training including those provided to professionals, it cannot be expected to provide sufficient basic subject knowledge nor can it be expected to build the necessary basic cross-cutting skills to qualify CHWs to deliver healthcare services.

The NC(V) Primary Health programme, by contrast, fulfils the 2018 WHO criteria. It is properly formulated in terms of content and articulation across levels and over time, giving students the opportunity to develop their abilities, and expand and deepen their knowledge. However, the legislative requirement that it should be a full-time offering is inappropriate for two fundamental reasons. Firstly, it reduces the practical learning potential that is derived from working and studying part-time. Secondly, and importantly, the target population expected to work as CHWs in community-based healthcare often cannot afford to be without income, because they are predominantly from poorer households, often have dependents and are expected to contribute financially to their families.

The WHO recommendation that the pre-service education curriculum should cover comprehensive healthcare and linkage to the healthcare system as well as cross-cutting and interpersonal skills was evident in NC(V) Primary Health programme. Content taught in the four vocational subjects provided sufficient theoretical depth to assist students to think critically, operate at a higher level of knowledge integration and apply what they have learnt. These, together with cross-cutting language, mathematics and life skills, contributed to individual personal growth and enriched learners’ record-keeping, communication, psychosocial and problem-solving abilities. Learners were provided with an essential understanding of confidentiality and healthcare service ethics. As evidenced in the training materials as well as the approaches to learning, theoretical knowledge was applied to practical and social competencies in order to enable learners to engage effectively with household members.^13^ The curriculum was developed by experts from various disciplines and delivered by well-oriented lecturers from different disciplines, including health sciences, education, and social and natural sciences. The use of an adult learning approach enabled learners to apply what they have learnt to their own lives as well as to the workplace or workplace scenarios.

The NC(V) Primary Health programme addressed the WHO recommendations regarding the use of blended learning and that training should be conducted in or near the community. The balance of theory and practice was more effective in the part-time offering because CHW students were able to practise what they had learnt and take their practical experience to the learning environment in a regular way. Even though the full-time offering of NC(V) Primary Health built in regular practical assignments, these were adversely influenced by the relationship between the DHET and NDoH.

## Conclusion

The South African government’s rPHC decision to extend primary healthcare to people in their homes and communities through the deployment of teams of CHWs is based on the need to engage more effectively with the quadruple disease burden, as well as the contribution that this cadre of healthcare workers makes to universal health coverage. It is well established, both in South Africa and internationally, that adequate, appropriate and effective education and training of healthcare professionals and workers, including CHWs, are critical determinants of the quality of healthcare services.^44^ This evaluation shows that in creating and implementing a standardised, curriculated national programme for CHW education to meet the NDoH’s rPHC 2010 requirements, the NC(V) Primary Health substantively follows 2018 WHO guidelines. Built on a foundation of developing CHW capacity to support generalist comprehensive care as part of an interdisciplinary, multi-professional service delivery, the programme structured theoretical and practical learning over time to ensure assimilation of content and its application in practice. The pilot part-time offering clearly showed a way to improve the value of the qualification for CHWs and the NDoH by allowing CHWs to work and study at the same time. Given the commonalities in healthcare service needs and healthcare system structure across the country, the NC(V) Primary Health programme demonstrates the value of a single, national, quality-assured qualification for CHWs. There is thus a case to be made for a pre-service NQF-aligned education curriculum to be set up nationally, which is supported and implemented at provincial level, and work-integrated training that is implemented at district level to focus on local needs.

Further research is needed into several key issues surrounding NDoH decisions and practices with respect to the NC(V) Primary Health offering, particularly delayed initiation, the parallel development of an NQF level 3 qualification by the Quality Council for Trades and Occupations (QCTO) and the failure to put in place processes to support graduate career pathing. The rationale behind the 2016 ministerial decision to terminate the NC(V) Primary Health has not been made public. That said, it needs to be reviewed, given the evidence from this evaluation that it is a productive, well-functioning, scope-aligned, standardised quality-assured learning programme that substantially addresses WHO guidelines and recommendations and NDoH training needs and many CHW learning expectations.

### Limitations

There are several limitations of this study. Fieldwork for the research was constrained by the disruptions caused by student protests over the poor articulation of the qualification with public health employment and the decision to suspend the programme. This affected the breadth and depth of the study, especially in terms of limiting the number of participants in the study. The DHET and NDoH officials were reluctant to engage with the research and it was only possible to access staff at two colleges as well as part-time lecturers and CHW-students, as permission to engage full-time students was not granted.
